# Using Mobile Health to Support Clinical Decision-Making to Improve Maternal and Neonatal Health Outcomes in Ghana: Insights of Frontline Health Worker Information Needs

**DOI:** 10.2196/12879

**Published:** 2019-05-24

**Authors:** Hannah Brown Amoakoh, Kerstin Klipstein-Grobusch, Diederick E Grobbee, Mary Amoakoh-Coleman, Ebenezer Oduro-Mensah, Charity Sarpong, Edith Frimpong, Gbenga A Kayode, Irene Akua Agyepong, Evelyn K Ansah

**Affiliations:** 1 Department of Epidemiology School of Public Health University of Ghana Accra Ghana; 2 Julius Center for Health Sciences and Primary Care University Medical Center Utrecht University Utrecht Netherlands; 3 Division of Epidemiology and Biostatistics School of Public Health, Faculty of Health Sciences University of the Witwatersrand Johannesburg South Africa; 4 Department of Epidemiology Noguchi Memorial Institute for Medical Research University of Ghana Accra Ghana; 5 La General Hospital Ghana Health Service Accra Ghana; 6 Regional Health Directorate Ghana Health Services Koforidua Ghana; 7 Dodowa Research Centre Ghana Health Service Accra Ghana; 8 Research and Development Division Ghana Health Service Accra Ghana; 9 Centre for Malaria Research University of Health and Allied Sciences Ho Ghana

**Keywords:** mHealth, maternal health, neonatal health, health care systems, developing countries, decision-making, information retrieval systems

## Abstract

**Background:**

Developing and maintaining resilient health systems in low-resource settings like Ghana requires innovative approaches that adapt technology to context to improve health outcomes. One such innovation was a mobile health (mHealth) clinical decision-making support system (mCDMSS) that utilized text messaging (short message service, SMS) of standard emergency maternal and neonatal protocols via an unstructured supplementary service data (USSD) on request of the health care providers. This mCDMSS was implemented in a cluster randomized controlled trial (CRCT) in the Eastern Region of Ghana.

**Objective:**

This study aimed to analyze the pattern of requests made to the USSD by health workers (HWs). We assessed the relationship between requests made to the USSD and types of maternal and neonatal morbidities reported in health facilities (HFs).

**Methods:**

For clusters in the intervention arm of the CRCT, all requests to the USSD during the 18-month intervention period were extracted from a remote server, and maternal and neonatal health outcomes of interest were obtained from the District Health Information System of Ghana. Chi-square and Fisher exact tests were used to compare the proportion and type of requests made to the USSD by cluster, facility type, and location; whether phones accessing the intervention were shared facility phones or individual-use phones (*type-of-phone*); or whether protocols were accessed during the day or at night (*time-of-day*). Trends in requests made were analyzed over 3 6-month periods. The relationship between requests made and the number of cases reported in HFs was assessed using Spearman correlation.

**Results:**

In total, 5329 requests from 72 (97%) participating HFs were made to the intervention. The average number of requests made per cluster was 667. Requests declined from the first to the third 6-month period (44.96% [2396/5329], 39.82% [2122/5329], and 15.22% [811/5329], respectively). Maternal conditions accounted for the majority of requests made (66.35% [3536/5329]). The most frequently accessed maternal conditions were postpartum hemorrhage (25.23% [892/3536]), *other conditions* (17.82% [630/3536]), and hypertension (16.49% [583/3536]), whereas the most frequently accessed neonatal conditions were prematurity (20.08% [360/1793]), sepsis (15.45% [277/1793]), and resuscitation (13.78% [247/1793]). Requests made to the mCDMSS varied significantly by cluster, type of request (maternal or neonatal), facility type and its location, *type-of-phone*, and *time-of-day* at 6-month interval (*P*<.001 for each variable). Trends in maternal and neonatal requests showed varying significance over each 6-month interval. Only asphyxia and sepsis cases showed significant correlations with the number of requests made (*r*=0.44 and *r*=0.79; *P*<.001 and *P*=.03, respectively).

**Conclusions:**

There were variations in the pattern of requests made to the mCDMSS over time. Detailed information regarding the use of the mCDMSS provides insight into the information needs of HWs for decision-making and an opportunity to focus support for HW training and ultimately improved maternal and neonatal health.

## Introduction

### Background

Weak health systems are a major barrier to achieving improved health outcomes in low- and middle-income countries [1]. It is therefore not surprising that many countries that could not attain the Millennium Development Goals (MDGs) 3 and 4 which targeted improvements in maternal, neonatal, and child health (MNCH), were from the parts of the globe with poorly developed health systems such as sub-Saharan Africa and Southern Asia [2]. As global efforts to improve MNCH intensifies through the Sustainable Development Goals (SDGs) 3.1 and 3.2 [3], health system strengthening has become imperative to attain these SDGs.

Among the many interventions currently being implemented to address MNCH challenges, mobile health (mHealth) interventions have been widely used in low- and middle-income countries [4] as a potential solution to maximize health worker (HW) impact, efficiency, and health outcomes [5,6] and improve service utilization [7]. Common areas of application of mHealth tools include point-of-care decision-making support, provider-to-provider communication, and data collection [4,8,9]. Though mHealth interventions are well received by HWs and the community [9-12], data about their effectiveness with regards to patient health outcomes, improved efficiency of health systems, or their use by HWs are limited [5,7-9,13,14].

Ghana, a sub-Saharan African country, reports unacceptably high maternal and neonatal deaths that fell short of the MDGs targets [2]. Ghana’s maternal mortality is presently estimated at 319 per 100,000 live births [15] and its neonatal mortality rate is 25 deaths per 1000 live births, with higher mortality rates being reported in rural areas of the country [16-19]. Though numerous training programs and maternal audits are performed in Ghana to improve the quality of MNCH services [20], health system constraints still remain. Health system constraints contributing to persistently high maternal and neonatal mortality in Ghana include cost, distance, availability of health facilities (HFs), attitude of nurses toward pregnant women [21,22], and nonadherence of HWs to clinical guidelines [23,24]. To address the constraint of poor adherence to clinical guidelines by HWs, we designed an mHealth intervention—a clinical decision-making support system (CDMSS) to facilitate easy access to maternal and neonatal guidelines for routine and emergency obstetric, antenatal, and neonatal care for frontline providers of maternal and neonatal care in Ghana [25].

### Description of the Intervention

This mHealth clinical decision-making support system (mCDMSS) consisted of 4 components: (1) Phone calls (to facilitate verbal communication between frontline health workers, FHWs), (2) SMS text messaging (short message service, SMS; to facilitate communication between FHWs during periods of nonsustained network connectivity), (3) Access to an unstructured supplementary service data (USSD) for standard emergency obstetric and neonatal protocols via SMS text messaging (to provide quick and easy access to the standard guidelines to maternal and neonatal health protocols in Ghana), and (4) Access to the internet (to facilitate access to health information that may not be found in the USSD protocols). All these components were embedded in a composite intervention on a project nonsmart mobile phone. The multifaceted nature of the mCDMSS was aimed to assure access to clinical decision-making support for HWs at all times following suggestions from FHWs for clinical decision-making support in a formative study [26]. Access to the USSD was considered to be the main intervention component. Health workers were expected to use the phones primarily to access neonatal and maternal health emergency protocols via the USSD and obtain additional support from colleagues and the internet via the other intervention components. The messages on the USSD were created by a team of FHWs, family physicians, obstetricians, and pediatricians in the Greater Accra Region, drawing on the Ghana’s Safe Motherhood protocols [27]. The development of the intervention was done using an iterative process that piloted and tested the USSD messages among FHWs in the Greater Accra Region to assure comprehension and appropriateness of the USSD messages. The USSD was designed such that new protocol requests needed to be initiated if a request session was terminated prematurely. FHWs, mainly midwives were provided with 312 dedicated nonsmart mobile phones to access the intervention. These phones were classified by the research team as shared facility phones if dedicated for shared-use by all providers of maternal and neonatal health care services in a HF or, as individual-use phones if dedicated to personal use of midwives. Each midwife at post in each HF during baseline assessment was provided with 1 mobile phone (individual-use phone) as they work closely with maternal and neonatal patients. FHWs were assumed to be familiar with the basic functioning of a mobile phone (making calls, texting, and accessing the internet) as documented in previous studies [28,29], so the training concerning the use of the mCDMSS focused on how to use the USSD. Navigation through the USSD has been demonstrated in Multimedia Appendix 1.

We tested the intervention in a cluster randomized controlled trial (CRCT) in the Eastern Region of Ghana. The CRCT has been described in detail elsewhere [25]. Vodafone Ghana, a telecommunication company, provided technical support for the mCDMSS and collected routine data regarding how the intervention was used throughout the intervention period.

### Study Objectives

The USSD component of the intervention explicitly and objectively provides insight into the information needs of FHWs. As details of protocols accessed from the USSD by FHWs are not known, we aimed to, first, describe the pattern of USSD protocol requests made by frontline providers of maternal and neonatal health services in district level HFs in the Eastern Region of Ghana and second, to examine the relationship between the patterns of requests made and the incidence of maternal and neonatal morbidity in HFs accessing the intervention.

## Methods

### Study Design and Sampling

This study was conducted within the context of the aforementioned CRCT, which aimed to assess the impact of the mCDMSS on institutional neonatal mortality in the Eastern Region of Ghana and comprised 16 districts randomized into 8 intervention and 8 control clusters. In a given cluster, all public and private HFs that work with the Ghana Health Service participated in the CRCT. We extracted all requests made to the USSD during the 18 months of intervention implementation (August 1, 2015 to January 31, 2017) from the USSD server of Vodafone Ghana; and all morbidity cases for the aforementioned timeframe for which requests were made, from the District Health Information Management System (DHIMS2) in Ghana. The DHIMS2 is a data recording, collection, collation, and analysis tool that hosts the entire national institutional health data of Ghana mainly from the public sector and a few private facilities [25].

This study was approved by the Ghana Health Service Ethics Review Committee before its commencement; study approval number GHS-ERC: 04/09/16.

### Data Collection

Before data extraction, phone numbers assigned to various users was collated such that each intervention user, the HF as well as the district (cluster) the user worked in, was documented and coded in Vodafone Ghana’s database. This ensured that requests made to the USSD could be traced back to the clusters, HFs, and FHWs using the phone. A total of 5 of the individual-use phones could not be traced back to the FHWs who received them as they were not signed for, and efforts to reach these numbers were futile. These 5 phone numbers were thus, not included in analysis. The USSD data were extracted monthly. Due to technical challenges at Vodafone Ghana, 22 days of data were lost during the first 6 months of the intervention. From the DHIMS2 database, maternal cases of postpartum hemorrhage (PPH), antepartum hemorrhage (APH), hypertensive disorders in pregnancy (HDP), and neonatal cases of prematurity, asphyxia, jaundice, cord sepsis, and sepsis occurring in the intervention period were extracted. In the DHIMS2, data captured regarding the aforementioned maternal cases cover hospital in-patients only. In the case of neonatal morbidity, the DHIMS2 captures data regarding neonatal cases of sepsis and prematurity at only hospital level, whereas neonatal cases of asphyxia, jaundice, and cord sepsis are captured as aggregate data for all types of HFs, that is, hospitals, health centers (HCs), and Community-based Health Planning and Services (CHPS) working with or within the Ghana Health Service. Due to challenges with the DHIMS2, some hospitals entered data concerning morbidities of interest that were not captured or could not be extracted from the DHIMS2 onto Excel spreadsheets that were given to the project team for analysis. The data entry in such situations was done by the hospital health information officers responsible for entering those data into the DHIMS2, and the data were validated by the head of the health information unit in these hospitals.

### Statistical Analysis

The data were checked for errors and exported from Microsoft Excel (Microsoft Corporation) to Stata version 13 (StataCorp LLC) for cleaning and analysis. We classified HFs into 2 groups of remote and nonremote areas based on access. Remote facilities were either located more than 30-min’ walk or more that 15-min motorbike ride from the main district township and had poor road access (uneven and untarred roads overcrowded with weeds and shrubs) leading to them. Nonremote HFs were either located within 30-min’ walk or 15-min motorbike ride from the main district township and had good road access leading to them. Due to the similarities in organizational structure, personnel and health services provided by CHPS, and maternity homes, requests from these 2 facility types were combined for analysis. Time of accessing the USSD was coded as day if requests were made from 6 am to 6 pm; all other time periods were coded night. Maternal morbidities— gestational hypertension, chronic hypertension, eclampsia, pre-eclampsia, and hypertensive encephalopathy were all classified as HDP. Placenta praevia and abruption were considered as APH, and retained placenta was considered as PPH as patients are usually hospitalized because of bleeding from these conditions. Unspecified cause of bleeding and vomiting were excluded during analysis. The Vodafone data were not corrected for the 22 days of missing data in the first 6 months of intervention implementation as the data were considered missing completely at random [30].

Descriptive analysis of requests made to the USSD server from clusters, HFs, *type-of-phone* (individual-use or shared-use), HF location, and *time-of-day* (explanatory variables) was done and expressed in numbers and percentages, first, as a combined 18-month data and then at 6-month intervals. Trends in maternal and neonatal requests were assessed. Chi-square and Fisher exact tests were applied to these analyses to assess the significance of the observed pattern of USSD requests. Morbidity from aforementioned cases of interest were estimated from the DHIMS2. The relationship between USSD requests and morbidity from cases for which requests were made was also estimated using Spearman correlation. All analyses were performed using Stata 13 statistical software and using 2-tailed tests at alpha=.05.

## Results

### User Statistics

A total of 74 HFs in all 8 intervention clusters were recruited into this study (Table 1). Each cluster included at least 1 district hospital but a varying mix of HCs and CHPS. In all, data from 307 mobile phones were analyzed; 74 were shared-use phones, whereas the rest were individual-use phones. At the end of the intervention period, a total of 5329 requests were made to the USSD. Of these requests, 2396 (44.96% [2396/5329]) were made during the first 6 months, 2122 (39.82% [2122/5329]) in the second 6 months, and 811 (15.22% [811/5329]) in the last 6 months. Throughout the intervention period, maternal requests (66.35% [3536/5329]) were made more frequently compared with neonatal requests (33.65% [1793/5329]). Requests per cluster ranged from 1167 (representing 21.90% [1167/5329] of total requests) to 403 (representing 7.56% [403/5329] of requests); the average request made per cluster was 667. All clusters made a request to the USSD. Of the 74 HFs (combined from all clusters), 72 accessed the USSD at least once during the intervention period. The 2 HFs that did not access the intervention included a privately owned maternity home that had no midwife at post throughout intervention implementation and a CHPS compound whose midwife shared during a routine supervisory visit by the research team that she trusted her competence in midwifery practice and so did not see the need to consult the USSD protocols. Among HFs, requests from hospitals declined from the first to the last 6 months, whereas requests from HCs and CHPS increased. Close to hundred percent (98.44% [2904/2950]) of all requests made from hospitals were with individual-use phones compared with the proportion of requests made with individual-use phones in HCs (52.87% [654/1237]) and CHPS (30.74% [351/1142]; *P*<.001).

At night, the proportion of requests made from HCs (27.81% [344/1237]) and CHPS (27.67% [316/1142]) was lower than the proportion of requests from hospitals (34.17% [1008/2950]; *P*<.001). There were similarities in the observed proportion of maternal protocols assessed by individual-use phones (65.49% [2560/3909]) and shared-use phones (68.73% [976/1420]; *P*=.03); and in the proportion of requests made at night by both phone types (27.62% [450/1630] for shared-use phones and 31.52% [1371/4350] for individual-use phones; *P*=.003). Shared-use phones were used more often in remote areas (78.24% [1111/1420]) compared with individual-use phones (11.49% [499/3909]) in accessing the intervention (*P*<.001). The frequency of shared-use phones accessing the intervention increased over time, whereas the frequency of individual-use phone decreased.

**Table 1:** Background characteristics of clusters.

Cluster^a^	Number of health facilities				Demographic location of health facilities, n (%)		Number of deliveries per midwife^b^	Proportion of shared phones received, n (%)
	Hospital^c^	HCs^d^	CHPS^e^	Maternity home^f^	Remote	Nonremote		
A	1	1	7	0	7 (78)	2 (22)	80.0	9^g^ (43)
B	1	7	3	0	6 (55)	5 (45)	130.4	11 (38)
C	3	2	9	1	6 (40)	9 (60)	99.0	15 (26)
D	2	3	3	0	5 (63)	3 (37)	94.8	8 (20)
E	1	3	2	0	3 (50)	3 (50)	107.6	6 (24)
F	1	8	1	1	4 (36)	7 (64)	101.6	11 (22)
G	3	2	0	1	1 (17)	5 (83)	75.0	6 (11)
H	1	3	3	1	4 (50)	4 (50)	96.4	8^g^ (26)

^a^Clusters have been named A-H for anonymity.

^b^Reference year is 2014.

^c^Includes both private and public hospitals.

^d^HCs: Health Centers.

^e^CHPS: Community-based Health Planning and Services.

^f^Includes only private maternity homes.

^g^This may differ slightly from the sum of the number of midwives in the cluster and the number of health facilities as 2 individual-use phones from these clusters could not be traced.

The proportion of maternal requests from remote (69.81% [1089/1560]) and nonremote areas (64.92% [2447/3769] as well as the proportion of requests made at night from remote (31.09% [485/1560]) and nonremote areas (31.39% [1183/3769]) were similar (*P*=.001 for request type, *P*=.046 for time of day requests were made). The frequency of remote areas accessing the intervention increased over time, whereas the frequency of nonremote areas decreased. Requests by clusters, HFs and their location, type of request (maternal or neonatal), *type-of-phone*, and *time-of-day* varied significantly at 6-month intervals during the intervention period (Table 2).

### Trends in Maternal Requests

Detailed analysis of maternal requests show that PPH protocols were accessed the most (27.22% [450/1653]) in the first 6 months, followed by *other conditions* protocols (16.76% [277/1653]) and HDP protocols (16.21% [268/1653]). This trend in requests was repeated in the second 6 months (PPH: 22.69% [300/1322], *other conditions*: 20.57% [272/1322], and HDP: 16.34% [216/1322]). In the last 6 months, HDP (17.7% [99/561]) was the second most accessed protocol after PPH (25.3 [142/561]), whereas APH and *other conditions* contributed 14.4% (81/561) each to requests made. Across clusters, this trend in maternal requests was significant at each 6-month interval (*P*<.001 for each timeframe). Across HFs, the trend of maternal requests aforementioned differed significantly only in the first and second timeframe (*P*=.04, *P*=.03, and *P*=.15, respectively); by *type-of-phone*, this trend varied at all 3 time points (*P*=.05, *P*=.01, and *P*<.001, respectively); and across HFs, maternal request trends differed in the third 6th month alone (*P*=.57, *P*=.42, and *P*=.001, respectively, for each timeframe).

**Table 2:** Distribution of unstructured supplementary service data requests at 6-monthly intervals.

Variable		First 6 months, frequency (%)	Second 6 months, frequency (%)	Third 6 months, frequency (%)	Total, frequency (%)	*P* value for χ^2^ test
**Cluster^a^**						
	A	244 (10.18)	216 (10.18)	198 (24.41)	658 (100.00)	<.001
	B	262 (10.93)	184 (8.67)	42 (5.18)	488 (100.00)	<.001
	C	406 (16.94)	311 (14.66)	97 (11.96)	814 (100.00)	<.001
	D	174 (7.26)	220 (10.37)	98 (12.08)	492 (100.00)	<.001
	E	173 (7.22)	153 (7.21)	77 (9.49)	403 (100.00)	<.001
	F	552 (23.04)	438 (20.64)	177 (21.82)	1167 (100.00)	<.001
	G	261 (10.89)	468 (22.05)	48 (5.92)	777 (100.00)	<.001
	H	324 (13.52)	132 (6.22)	74 (9.12)	530 (100.00)	<.001
**Type of request**						
	Maternal care	1653 (68.99)	1322 (62.30)	561 (69.17)	3536 (100.00)	<.001
	Neonatal care	743 (31.01)	800 (37.70)	250 (30.83)	1793 (100.00)	<.001
**Type of facility**						
	Hospitals	1563 (65.23)	1069 (50.38)	318 (39.21)	2950 (100.00)	<.001
	HCs^b^	418 (17.45)	587 (27.66)	232 (28.16)	1237 (100.000	<.001
	CHPS^c^ and maternity homes	415 (17.32)	466 (21.96)	261 (32.18)	1142 (100.00)	<.001
**Type of phone**						
	Individual-use	1921(80.18)	1531 (72.15)	457 (56.35)	3903 (100.00)	<.001
	Shared-use	475 (19.82)	591 (27.85)	354(43.65)	1420 (100.00)	<.001
**Demographic location**						
	Nonremote	1906 (79.55)	1435 (67.62)	457 (56.35)	3769 (100.00)	<.001
	Remote	490 (20.45)	687 (32.38)	354 (43.65)	1560 (100.00)	<.001
**Time of day**						
	Day	1573 (65.65)	1526 (71.91)	562 (69.30)	3661 (100.00)	<.001
	Night	823 (34.35)	596 (28.09)	249 (30.70)	1668 (100.00)	<.001

^a^Clusters have been named A-H for anonymity.

^b^HCs: Health Centers.

^c^CHPS: Community-based Health Planning and Services.

**Figure 1:**
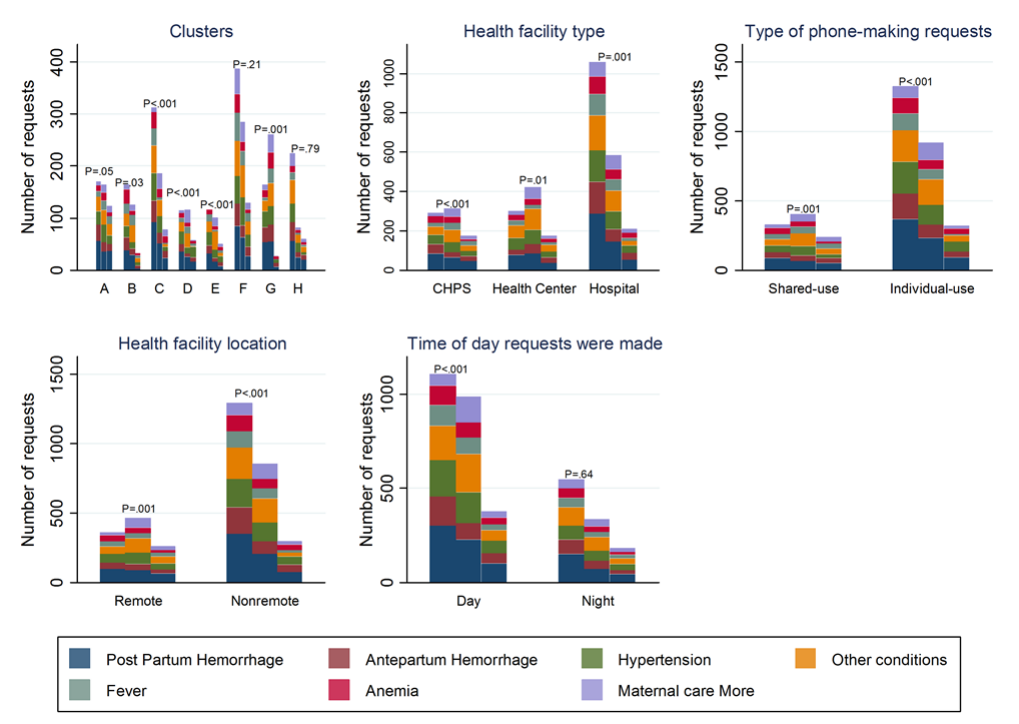
Maternal requests at 6-month interval by cluster, health facility type and location, phone type, and time of day requests were made. P values are chi-square tests or Fisher exact test comparing requests within each subcategory of each explanatory variable at 6-month intervals. Clusters have been labeled A-H for anonymity; CHPS: Community-based Health Planning and Services.

There was no variation in maternal requests trends by *time-of-day* requests were made at 6-month intervals (*P*=.16, *P*=.58, and *P*=.93, respectively). Detailed analysis of maternal requests pertaining to *other conditions* shows that hyperemesis was the most frequently requested protocol accounting for 26.3% (47/179) and 37.4% (70/187) of requests in the first and second 6 months, respectively. This was followed by fetal distress, which accounted for 18.4% (33/179) and 13.9% (26/187) of requests and premature rupture of membranes for gestation <37 weeks, which accounted for 17.3% (31/179) and 13.9% (26/187) of requests for *other conditions* for the same timeframe. In the third 6 months, cord prolapse, hyperemesis, and premature rupture of membranes for gestation <37 weeks accounted for 28% (15/54), 28% (15/54), and 19% (10/54) of *other conditions* request, respectively. Figure 1 describes in detail the pattern of maternal requests made by the clusters, HFs and their location, *type-of-phone*, and *time-of-day* for each 6-month period. Overall, there was a 20.02% (331/1653) and a 57.56% (761/1322) decline respectively, in the number of maternal requests made from the first to the second 6 months and from the second to the third 6 months of intervention implementation.

### Trends in Neonatal Requests

**Figure 2:**
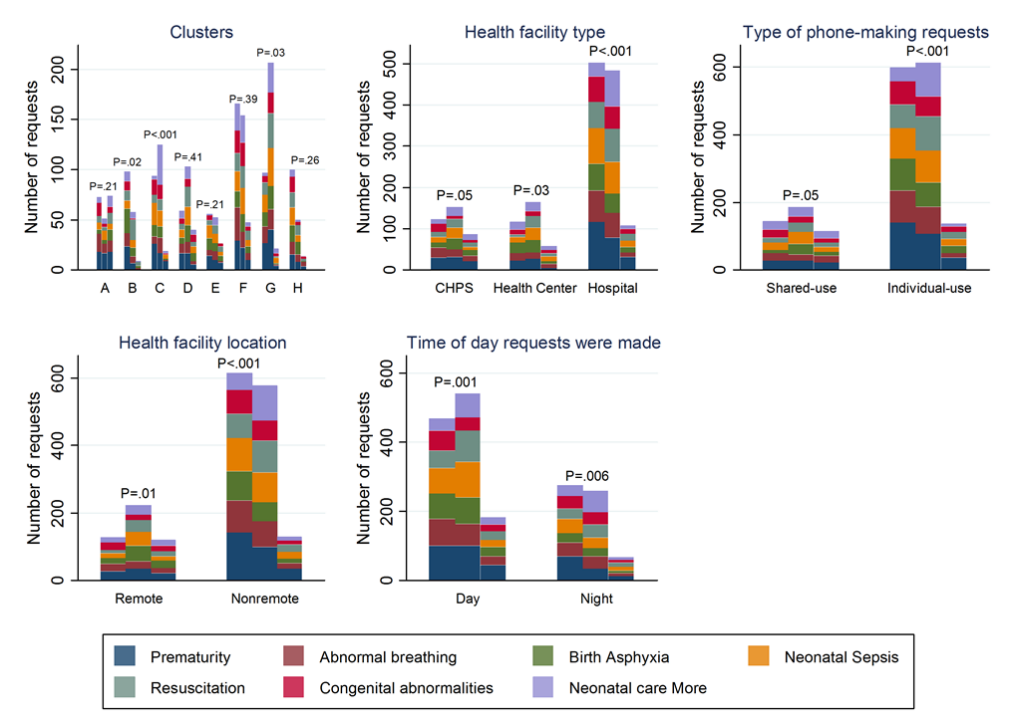
Neonatal requests at 6-month interval by cluster, health facility type and location, phone type, and time of day requests were made. P values are chi-square tests or Fisher exact test comparing requests within each subcategory of each explanatory variable at 6-month intervals. Clusters have been labeled A-H for anonymity; CHPS: Community-based Health Planning and Services.

Trends in neonatal requests show that prematurity protocols were accessed the most (22.6% [168/743]) in the first 6 months, followed by *abnormal breathing* protocols (15.8% [117/743]) and neonatal sepsis protocols (16.2% [113/743]). In the second 6 months, prematurity was most requested (16.9% [135/800]), followed by neonatal sepsis (16.38% [131/800]) and then *neonatal more* (16.3% [130/800]). In the last 6 months, frequently requested protocols were prematurity, resuscitation, and asphyxia in descending order of 22.8% (57/250), 14.8% (37/250), and 14.0% (35/250), respectively. Across clusters, this trend in neonatal requests was significantly different during the first and second 6 months of intervention implementation (*P*<.001 in each interval and *P*=.12 in the third 6 months). Across HFs, this trend of neonatal requests was again significantly different during the first 6 months (*P*=.001, *P*=.07, and *P*=.15, respectively, per interval); by *type-of-phone*, the observed trend aforementioned varied significantly during the first 6 months (*P*<.001, *P*=.38, and *P*=.07, respectively, per timeframe); by HF location and *time-of-day*, requests varied during the second 6 months only (*P* values for location type=.31, *P*=.001, and *P*=.13, respectively; *P* values for *time-of-day* analysis=.20, <.001, and .78, respectively). Detailed analysis of *neonatal more* requests show that 55.4% (72/130) of requests concerned neonatal seizures and the rest concerned birth trauma. Figure 2 describes in detail the pattern of neonatal requests made by the clusters, HFs and their location, *type-of-phone*, and *time-of-day* for each 6-month period. Overall, there was a 7.7% (57/743) increase and then a 68.8% (550/800) decline, respectively, in the number of neonatal requests made from the first to the second 6 months and from the second to the third 6 months of intervention implementation.

### Correlation Between Requests Made and Incidence of Cases

Generally, the number of maternal and neonatal cases exceeded the number of requests made except in the case of PPH. The correlation between requests made and actual number of cases recorded in HFs ranged from weak to strong positive and negative correlations. Spearman correlation was, however, significant for only asphyxia (Spearman rho=.44; *P*<.001) and sepsis cases (Spearman rho=.79; *P*=.03). Table 3 details the correlation coefficients for all outcomes of interest.

**Table 3:** Correlation between requests made to the intervention and actual number of cases recorded in health facilities.

Type of case		Number of requests^a^ (%)	Number of cases (%)	Spearman rho	*P* value
**Maternal^b^**					
	Antepartum hemorrhage	231 (51.7)	242 (100.0)	.05	.90
	Postpartum hemorrhage	438 (49.1)	298 (100.0)	–.03	.93
	Hypertension	267 (45.8)	1339 (100.0)	–.32	.41
**Neonatal**					
	Asphyxia	320 (100.0)	2004 (100.0)	.44	<.001
	Jaundice	15 (100.0)	158 (100.0)	.18	.12
	Cord sepsis	6 (100.0)	63 (100.0)	–.07	.57
	Sepsis^b^	124 (40.4)	185 (100.0)	.79	.03
	Prematurity^b^	208 (57.9)	831 (100.0)	–.22	.58

^a^Represents the proportion of requests from only health facilities included in analysis.

^b^Represents hospital level data excluding 3 of the 12 hospitals in the intervention arm. Two of these 3 hospitals are in the same district and are privately owned; data from hospitals excluded were unavailable to researchers as of time of data analysis (August 2018).

## Discussion

### Principal Findings

This study describes the pattern of requests made to a SMS text messaging–based mobile CDMSS by FHWs providing maternal and neonatal health services in Ghana. We assessed the relationship between protocol requests made and types of maternal and neonatal morbidities for which requests were made. All clusters accessed the intervention, which is consistent with known findings of general acceptability of mhealth interventions among HWs and communities [9-12]. Maternal protocols were requested for more often than neonatal protocols, suggesting differences in information needs among FHWs with regards to maternal and neonatal care. Such differences in information needs was previously documented among community-level HWs in Nepal who seemed to be more knowledgeable in neonatal than maternal care matters [31]. This observation could also be a reaction of FHWs to the extensive maternal death audits conducted in Ghana [20]. Neonatal deaths, on the other hand, have not received such attention.

The high number of requests for protocols of PPH, HDP, prematurity, and sepsis in our study reflects the global and local trend in maternal and neonatal morbidity where these morbidities top the list [2,32-34]. This observation emphasizes the persistence of these morbidities in low-resource settings and the consequent need for health system strengthening in this regard and focus on these areas during HW training.

Within clusters, PPH and prematurity protocols were most commonly requested, suggesting that FHWs in the different clusters have a common information gap regarding these 2 morbidities that was bridged by this intervention. Differences in requests by category of HFs appears to reflect the differences in information needs of FHWs at the different levels of health care [35]. Surprisingly, the majority of requests emanated from hospitals where one would assume resource availability to be higher. The higher number of requests from hospital FHWs could indicate an unmet need for clinical decision-making support that is action-oriented even at higher levels of the health system. D’Adamo and his colleagues made similar findings of a near or complete lack of access to current useful information for district- and community-level HWs in their study [35]. It is striking that the trend in both maternal and neonatal requests did not differ significantly by HF location at all 3 time points in our study. A plausible explanation for this observation is similarities in competencies of FHWs in both remote and nonremote settings. This may be particularly true as in the Ghana Health Service, HWs may be freely transferred from 1 HF type to another. Similarities in FHW competencies across HFs may explain the absence of differences in request trends by the *time-of-day* requests were made. Detailed information about the FHWs who utilized the intervention could have provided more information regarding this analysis but was not collected in this study. The higher proportion of requests made by nonremote areas compared with remote areas is most likely because of the higher proportion of individual-use phones in nonremotes areas where there are generally higher numbers of midwives. Hence, a collinear relationship between requests made by *type-of-phone* and HF location can be observed. However, it is remarkable that nearly all requests from hospitals were made with individual-use phones implying a near absolute redundancy of shared-use phones in hospitals. This observation suggests that FHWs who were given the project phones are probably the same and only people who used the intervention in hospitals. Lack of knowledge transfer concerning the availability and use of the intervention with other FHWs who missed the project team’s training sessions, the practice of keeping project phones under lock and key in senior colleagues offices, and the use of project phones as though they were individual-use phones by HWs who received these phones on behalf of the HFs, as documented in a study to understand how and why the intervention was used [36], could explain the low number of USSD requests by hospital shared-use phones. These observations are common health system challenges in low-resource settings that need to be addressed as not all HWs may attend the various training programs constantly organized for staff, and scarce resources have to be shared.

The setup of our intervention database is unique and allowed for in-depth analysis of requests made to the USSD by individual users unlike previous work [11]. Our study shows varying pattern of requests for emergency protocols across and within clusters, HFs and their location*, type-of-phone*, and *time-of-day* and type of request made at all 3 time points considered in this study. This reflects the dynamic nature of information needs of FHWs. Such dynamism has been reported [10,37,38] and is important to take into account in the design and maintenance of CDMSS [10,12,37,38] as well as training for FHWs.

The intervention phones were predominantly used for voice calls (64%), followed by data (28%), SMS text messaging (5%), and USSD to access protocols (2%), respectively [36]. Differential baseline technological literacy among FHWs may have impacted the use of the different intervention components [36]. The declined usage of the USSD over time can be explained by the so-called *novelty effect* associated with mhealth interventions [37,39-42]. Novelty effect is the tendency for performance to initially improve when new technology is instituted, not because of any actual improvement in learning or achievement, but in response to increased interest in the new technology [43]. However, the *novelty-effect* alone cannot be considered as the reason for the much lower number of requests made in the last 6 months of the study. Another possible explanation for this phenomenon may be *testing effect in learning* (that long-term memory is often increased when some of the learning period is devoted to retrieving the to-be-remembered information [44]). The much lower usage of the USSD in the last 6 months is most likely because of conversion of USSD protocols into tacit knowledge of FHWs [36]. Availability of specialist obstetricians, doctors, and senior midwives in hospitals and the need to conform to instructions from superior colleagues (eg, doctors) can also explain this finding, particularly in hospitals where the observed decline in requests was highest [36]. Conflict from overreliance on CDMSS [37] by users of the CDMSS and provider knowledge and experience from nonusers of the CDMSS may lead to abandonment of the CDMSS in resolving such conflicts with the mindset that critical thinking of the human mind must not be taken over by a CDMSS [37]. Where there is a disconnect between protocols and the reality on the ground (such as lack of equipment), HW may also decide not to access electronic resources [10]. Technical and supervisory support to motivate users may also play a role in the decline in requests observed [12,36], and thus, this observation warrants further probing.

The moderate to strong correlation between the number of sepsis and asphyxia requests suggest that FHWs actually encountered these cases and used these protocols in their decision-making for these morbidities. The converse may be true where weak and negative associations are observed; exploration of the USSD protocols to satisfy FHW curiosity and mobile network problems [11,45,46] necessitating that FHWs send multiple requests may explain these weak and negative associations.

### Limitations

Though our study highlights important patterns of use of a SMS text messaging–based CDMSS, the use of information accessed in the care of patients or clients is undetermined in this study. Though this limitation is inherent in the design of this study, this study provides much needed insights as to how an mHealth SMS text messaging–based CDMSS functioned in a low-resource setting and quantifies the information needs of FHWs providing maternal and neonatal health care in this type of setting. Insight into the information needs of FHWs can inform the design of interventions mHealth (or otherwise) to meet these needs.

### Conclusions

This study demonstrates that health care providers of maternal and neonatal health services in Ghana readily use a mobile SMS text messaging–based CDMSS in their clinical decision-making. These FHWs used the mHealth tool to request emergency protocols depending on their information needs, which varied across and within clusters, HFs and their location, and with time. Thus, the information needs of HWs is not static but continues to change over time requiring health system strengthening strategies that take cognizance of this dynamism. Mechanisms to sustain utilization of similar mHealth CDMSS interventions must be designed to suit relevant context if such interventions will be up-scaled as health system strengthening strategies in future.

## Appendix

Multimedia Appendix 1 Steps to request for emergency maternal or neonatal protocols.

## Abbreviations

APH: antepartum hemorrhage
CDMSS: clinical decision-making support system
CHPS: Community-based Health Planning and Services
CRCT: cluster randomized controlled trial
DHIMS2: District Health Information Management System 2
FHWs: frontline health workers
HCs: health centers
HDP: hypertensive disorders in pregnancy
HFs: health facilities
HWs: health workers
mCDMSS: mHealth clinical decision-making support system
MDGs: Millennium Development Goals
mHealth: mobile health
MNCH: maternal, neonatal, and child health
PPH: postpartum hemorrhage
SDGs: Sustainable Development Goals
SMS: short message service
USSD: unstructured supplementary service data
