# Supplementary material for: Using Mobile Health to Support Clinical Decision-Making to Improve Maternal and Neonatal Health Outcomes in Ghana: Insights of Frontline Health Worker Information Needs
Source: JMIR Mhealth Uhealth. 2019 May 24;7(5):e12879. doi: 10.2196/12879 (PMC6555115; doi:10.2196/12879)
Supplement: Multimedia Appendix 1 [file mhealth_v7i5e12879_app1.pdf]

Multimedia appendix: Steps to request for emergency maternal or neonatal protocols

Step 1: Dial short code (*9191#)

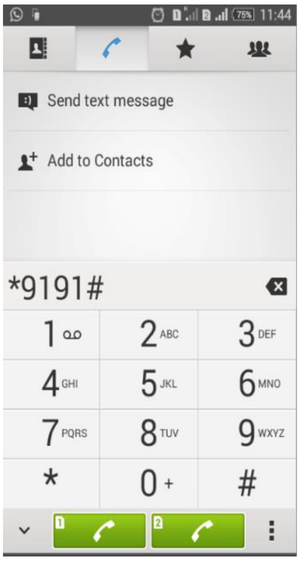

Step 2: Choose protocol type (maternal or neonatal)

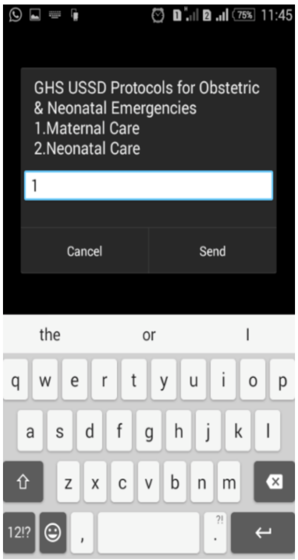

Step 3: Choose type of maternal protocol of interest

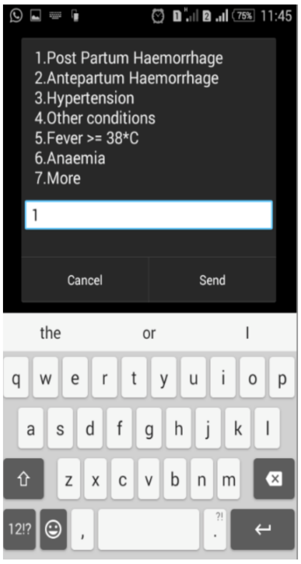

Step 4: Choose suspected course of maternal morbidity

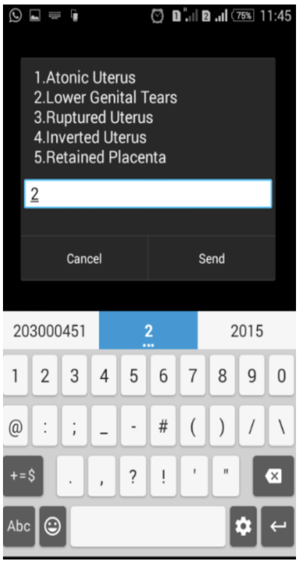

Step 5: Read guidelines for protocol assessed

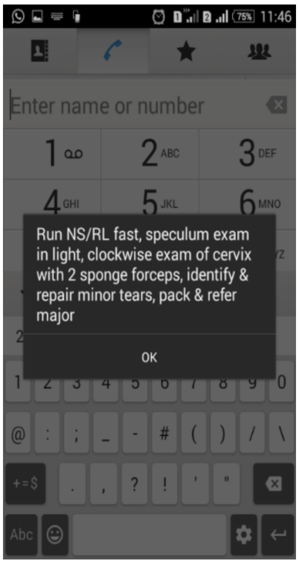

Steps 1 and 2 must always be performed. The number of remaining steps to finally receiving protocols may differ depending on the pathway that must be navigated to reach the protocol for a specified maternal or neonatal morbidity.
